# Baseline characteristics of myopic choroidal neovascularization in patients above 50 years old and prognostic factors after intravitreal conbercept treatment

**DOI:** 10.1038/s41598-021-86835-6

**Published:** 2021-04-01

**Authors:** Hai-Yan Wang, Meng-Zhang Tao, Xi-Xi Wang, Man-Hong Li, Zi-Feng Zhang, Dong-Jie Sun, Jin-Ting Zhu, Yu-Sheng Wang

**Affiliations:** 1 Department of Ophthalmology, Xijing Hospital, Xi’an, 710032 Shaanxi China; 2 Xi’an People’s Hospital (Xi’an Fourth Hospital), Shaanxi Eye Hospital, Affiliated Hospital of Northwestern Polytechnical University, Xi’an, 710001 Shaanxi China; 3 Department of Mathematics and Statistics, University of Arkansas at Little Rock, Little Rock, AR 72204 USA

**Keywords:** Diseases, Health care

## Abstract

To investigate the influence of age on the function and morphology of patients with myopic choroidal neovascularization (mCNV) and to evaluate the effect and prognostic factors of recurrence of Conbercept treatment on mCNV patients over 50 years. A total of 64 patients (64 eyes) with mCNV were enrolled in this retrospective study. The differences in baseline best-corrected visual acuity (BCVA) and morphological features on imaging between the younger group (˂ 50 years) and the older group (≥ 50 years) were analyzed. Of all, 21 eyes of 21 mCNV patients aged over 50 years who received Conbercept injection were further analyzed. Between the younger and the older group, significant differences were shown in mean BCVA (0.58 ± 0.28 vs 0.77 ± 0.31), subfoveal choroidal thickness (SFCT) (108.17 ± 78.32 μm vs 54.68 ± 39.03 μm) and frequency of vitreoretinal interface abnormalities (VIA) (2 vs 13), respectively (*P* < 0.05). After treated with Conbercept, the mean BCVA of 21 older mCNV patients increased from 0.83 ± 0.30 at baseline to 0.49 ± 0.24 at one year. Baseline BCVA, external limiting membrane damage, CNV area and CNV location correlated with the visual acuity at the 1-year follow-up. There were 7 (33.3%) recurrent cases during the follow-up and the risk of recurrence in patients with baseline central macular thickness (CMT) ≥ 262.86 μm was 14 times greater than that of patients with CMT < 262.86 μm. The risk of recurrence increased 1.84 times for every 100-μm increment in the CMT. Patients over 50 years with mCNV had a worse BCVA, thinner choroid, and higher risk of VIA than young mCNV patients. The standard Conbercept treatment strategy was safe and effective in mCNV patients over 50 years. As patients over 50 years with a greater CMT have a high risk of recurrence, more attention should be paid on these patients by following them up closely.

## Introduction

Pathologic myopia (PM) affects more than 3% of the world's population and its incidence is increasing with time^1^. Choroidal neovascularization secondary to PM leads to irreversible vision loss^2^. However, compared to younger PM patients, the older PM patients have a higher incidence of myopic choroidal neovascularization (mCNV) and a lower visual acuity at baseline and after the 5-year follow-up during the natural course^3,4^. Besides, the diagnosis of mCNV in PM patients over 50 years old should be differentiated from wet age-related macular degeneration (wAMD) due to the similar onset age and dissimilar treatment regimen and prognosis. Therefore, it is important to pay more attention to the diagnosis and treatment of mCNV in the older PM patients.

Currently, intravitreal injection of anti-vascular endothelial growth factor (VEGF) drugs has become the standard-of-care and the first-line treatment for mCNV^5–8^. Although the predictors of vision prognosis have been reported, including the baseline visual acuity, size and location of CNV, subfoveal choroidal thickness (SFCT), the prognosis and recurrence of mCNV in older patients are not fully understood. In this study, we aimed to describe the functional and morphologic characteristics of mCNV in patients over 50 years old and to evaluate the baseline predictors of Conbercept (Chengdu Kanghong Biotech Co., Ltd., Sichuan, China) efficacy and of recurrence in the treatment of older mCNV patients.

## Materials and methods

### Subjects

A retrospective, observational study was conducted at the Department of Ophthalmology, Xijing Hospital and Shaanxi Eye Hospital. A total of 64 patients with newly diagnosed CNV secondary to PM were enrolled in the study from September 2015 to May 2019. The study was conducted in accordance with the Declaration of Helsinki. And the study was approved by the Ethics Committee of XiJing Hospital. Informed consent was obtained from all the patients before inclusion.

Inclusion criteria for the observational study were as below: (1) axial length ˃26.0 mm or spherical equivalent refractive error ˂ − 6.0 diopter, and a characteristic pathologic fundus change (including lacquer cracks, chorioretinal atrophy, posterior staphyloma, or atrophic patches, etc.); (2) active CNV lesions in the macular area; (3) newly diagnosed CNV; and (4) no prior treatment with the photodynamic therapy (PDT) or anti-VEGF drugs.

Patients with the following conditions were excluded: (1) CNV secondary to other eye diseases (including wAMD, idiopathic CNV, etc.); (2) significant diseases of the respiratory, cardiovascular, digestive, urinary, or other systems inappropriate for anti-VEGF treatment; (3) any concomitant ocular diseases other than myopia and myopia-related retinopathy that might affect imaging quality or visual outcome in the study eye; and (4) cataract surgery during the follow-up.

Active mCNV was defined as any one of the following with a recent worsening of vision: (1) intra-retinal fluid (IRF) or sub-retinal fluid (SRF) on spectral domain optical coherence tomography (SD-OCT); (2) a hyperreflective lesion with fuzzy borders observed on SD-OCT; (3) appearance of new bleeding in the macular area; (4) leakage on the fundus fluorescein angiography or indocyanine green angiography (FFA/ICGA).

Furthermore, of all the patients enrolled above, patients over 50 years old who received Conbercept injection and were followed-up for one year were included in the subsequent Conbercept study. Thus, a total of 21 eyes of 21 mCNV patients were further included. The other eyes of the patients in the older group of the observation study were excluded because they either accepted another anti-VEGF drug other than Conbercept, or were not willing to be followed for one year as required by our protocol.

### Methods

Totally, 64 eligible patients were enrolled and divided into two groups according to their age, i.e. the younger group (˂50 years old) and the older group (≥ 50 years old). Demographic and baseline characteristics, especially morphologic features, such as IRF, SRF, ellipsoid zone (EZ) integrity, external limiting membrane (ELM) integrity, central macular thickness (CMT), SFCT, and vitreomacular interface abnormities (VIA), were analyzed and compared between both groups.

The total 64 mCNV patients in our study were either treated by anti-VEGF injection including but not limit to Conbercept or chose observation, and followed up according to our clinic protocol.

In the Conbercept study, a total of 21 eyes of mCNV patients in the older group were given one intravitreal injection of 0.5 mg Conbercept at baseline, and then followed-up and injected monthly until inactivity of the CNV. Immediately no active CNV was appreciated, treatment was discontinued and subsequent monthly follow-up was performed continuously for three months. Thereafter, patients were followed-up by monthly telephone interviews and ophthalmological visits every three months. Conbercept were reinjected intravitreally once an active CNV was detected, and the treatment regimen as well as follow-up was the same as the protocol above. The number of injections administered during the one-year follow-up period and the time between the initial injection and the CNV recurrence were recorded.

### Statistical analysis

The differences in baseline BCVA and the morphological features on imaging between the younger and older groups were analyzed using the T-test and chi-square test or Fisher’s exact test. Pearson correlation coefficient was calculated to evaluate the correlation between age and baseline BCVA, as well as age and baseline SFCT.

In the older group, eyes that received the Conbercept injection and completed the one-year follow-up visit were included in the efficacy and prognostic analysis. Thus, we measured the changes in visual acuity and CMT at the follow-up time points (1st month, 3rd month, 6th month, and 12th month). T-test and repeated measures ANOVA were used to evaluate the BCVA and CMT during follow-up.

Prognostic factors including demographic and functional factors as well as biomarkers on FA/OCT imaging for visual outcomes at 12 months were analyzed using univariate and multivariate linear regression analysis. Predictors for visual improvement at the 1st, 3rd, 6th, and 12th months were analyzed using the linear mixed-effects model. Predictors of recurrence were analyzed using the COX proportional hazards model. Statistical Package for Social Sciences (version 23.0; SPSS, Chicago, IL, USA) was used for statistical evaluation. The significance was determined based on an alpha error equal to or smaller than 5%.

## Results

### Demographic and baseline characteristics of the mCNV eyes

A total of 64 eyes of 64 patients (43 females and 21 males) with a mean age of 51.03 ± 13.56 years (range, 22–76 years) were included in the observational study. All patients were divided into the younger group (age ˂ 50 years, N = 25) with a mean age of 37.56 ± 7.78 years and the older group (age ≥ 50 years, N = 39) with a mean age of 59.67 ± 8.40 years. The demographic and morphologic characteristics of all the patients enrolled are described in Table 1.

**Table 1:** Demographic and baseline characteristics of mCNV eyes and comparison between the younger group (˂50 years) and older group (≥ 50 years).

Characteristics	Whole cohort (N = 64)	Age < 50 years (N = 25)	Age ≥ 50 years (N = 39)	*P*
**Gender**				
Male	21 (32.8%)	11 (44%)	10 (25.6%)	0.174
Female	43 (67.2%)	14 (56%)	29 (74.4%)	
**Age**				
Mean ± SD	51.03 ± 13.56	37.56 ± 7.78	59.67 ± 8.40	0.001
Range	(22–76)	(22–48)	(50–76)	
**Axial length**				
Mean ± SD	29.14 ± 1.73	29.29 ± 1.83	29.04 ± 1.67	0.634
Range	(26.61–32.75)	(26.72–32.75)	(26.61–32.40)	
**Baseline BCVA (logMAR)**				
Mean ± SD	0.69 ± 0.31	0.58 ± 0.28	0.77 ± 0.31	0.018
Range	(0–1.30)	(0–1.10)	(0.30–1.30)	
**Baseline CMT**				
Mean ± SD	274.70 ± 148.95	274.16 ± 100.11	275.05 ± 174.50	0.982
Range	(41–883)	(41–424)	(49–883)	
**Baseline SFCT**				
Mean ± SD	75.39 ± 62.66	108.17 ± 78.32	54.68 ± 39.03	0.004
Range	(16–340)	(28–340)	(16–228)	
**Baseline CNV area**				
Mean ± SD	1.05 ± 1.73	1.03 ± 1.93	1.06 ± 1.62	0.940
Range	(0.04–8.19)	(0.04–7.97)	(0.05–8.19)	
**CNV location**				
Subfoveal	55 (85.9%)	21 (84%)	34 (87.2%)	1
Juxtafoveal	9 (14.1%)	4 (16%)	5 (12.8%)	
**IRF**				
Present	8 (12.5%)	22 (88%)	34 (87.2%)	1
Absent	56 (87.5%)	3 (12%)	5 (12.8%)	
**SRF**				
Present	25 (39.2%)	11 (44%)	21 (53.8%)	0.609
Absent	39 (60.9%)	14 (%)	18 (46.2%)	
**EZ integrity**				
Present	5 (7.8%)	1 (%)	4 (10.5%)	0.609
Disrupt	48 (75.0%)	19 (%)	29 (76.3%)	
Absent	10 (15.6%)	5 (%)	5 (13.2%)	
**ELM integrity**				
Present	27 (42.2%)	10 (40%)	17 (44.7%)	0.925
Disrupt	31 (48.4%)	13 (52%)	18 (47.4%)	
Absent	5 (7.8%)	2 (8%)	3 (7.9%)	
**Vitreomacular abnormities**				
Absent	49 (76.6%)	23 (92%)	26 (66.7%)	0.028
VMA	4 (6.3%)	2 (8%)	2 (5.1%)	
VMT	5 (7.8%)	0	5 (12.8%)	
Foveoschisis	6 (9.4%)	0	6 (15.4%)	

*BCVA* best-corrected visual acuity, *CMT* central macular thickness, *CNV* choroidal neovascularization, *ELM* external limiting membrane, *IRF* intraretinal fluid, *EZ* ellipsoid zone, *logMAR* logarithm of the minimum angle of resolution, *SD* standard deviation, *SFCT* subfoveal choroidal thickness, *SRF* subretinal fluid, *VMA* vitreomacular adhesion, *VMT* vitreomacular traction.

There were significant differences in baseline BCVA (0.58 ± 0.28 vs 0.77 ± 0.31, *t* = 2.439, *P* = 0.018) and SFCT (54.68 ± 39.03 vs 108.17 ± 78.32 μm, *t* = –3.11, *P* < 0.01) between the two groups (the older group vs the younger group). Additionally, a linear negative correlation was shown between baseline BCVA and age (*r*^2^ = 0.121, *P* = 0.005). Similarly, a linear negative correlation was observed between the baseline SFCT and age (*r*^2^ = 0.216, *P* < 0.001).

In terms of VIA, only 2 patients in the younger group have a vitreomacular adhesion (VMA). Among the 39 patients in the older group, there were 13 eyes with VIA including 2 eyes with VMA, 5 eyes with vitreomacular traction (VMT), and 6 eyes with foveoschisis. Our data identified that more VIA was visualized in the older group than in the younger one by Fisher’s exact test (*P* = 0.032). No significant differences were found in the axial length, CMT, IRF, SRF, EZ integrity, ELM integrity, and CNV area (Table 1) between the younger and older groups.

### Baseline features and outcome of older mCNV patients treated with intravitreal Conbercept

In the older group, a total of 21 eyes of 21 patients (14 females and 7 males) with a mean age of 62.05 ± 8.66 years (range, 50–76 years) who completed 12 months of follow up were included in Conbercept treatment study. The mean axial length of these eyes was 29.50 ± 1.39 mm (27.34–32.40 mm).

Among the patients, there were 11 (52.4%) patients suffering mCNV in the right eyes and 10 (47.6%) patients in the left eyes, 16 (76.2%) eyes with subfoveal CNV, and 5 (23.8%) eyes with juxtafoveal CNV. IRF was discovered in 2 (9.5%) eyes and SRF in 10 (47.6%) eyes. ELM was intactly present in 8 (38.1%) eyes, disrupted in 12 (57.1%) eyes, and absent in 1 (4.8%) eye. EZ was intactly present in 1(4.8%) eye, disrupted in 18 (85.7%) eyes, and absent in 2 (9.5%) eyes. In addition, there were 7 (33.3%) eyes with VIA. Baseline demographic and clinical characteristics on imaging were described in Table 2.

**Table 2:** Baseline characteristics of mCNV eyes in patients over 50 years old receiving Conbercept treatment.

Characteristics	Value
**Gender, n (%)**	
Male	7 (33.3%)
Female	14 (66.7%)
**CNV area (mm**^**2**^**)**	
Mean ± SD (range)	1.41 ± 2.27 (0.03–8.19)
**CNV location, n (%)**	
Subfoveal	16 (76.2%)
Juxtafoveal	5 (23.8%)
**Lacquer cracks, n (%)**	
Absent	5 (23.8%)
Linear	5 (23.8%)
Stellate	11 (52.4%)
**IRF, n (%)**	
Present	2 (9.5%)
Absent	19 (90.5%)
**SRF, n (%)**	
Present	10 (47.6%)
Absent	11 (52.4%)
**Vitreomacular abnormities, n (%)**	
Absent	14 (66.7%)
VMA	1 (4.8%)
VMT	3 (14.3%)
Foveoschisis	3 (14.3%)
**EZ integrity, n (%)**	
Present	1 (4.8%)
Disrupt	18 (85.7%)
Absent	2 (9.5%)
**ELM integrity, n (%)**	
Present	8 (38.1%)
Disrupt	12 (57.1%)
Absent	1 (4.8%)

*BCVA* best-corrected visual acuity, *CNV* choroidal Neovascularization, *IRF* intraretinal fluid, *SRF* subretinal fluid, *SFCT* subfoveal choroidal thickness, *VMA* vitreomacular adhesion, *VMT* vitreomacular traction, *EZ* ellipsoid zone, *ELM* external limiting membrane, *logMAR* logarithm of the minimum angle of resolution, *SD* standard deviation.

**Figure 1:**
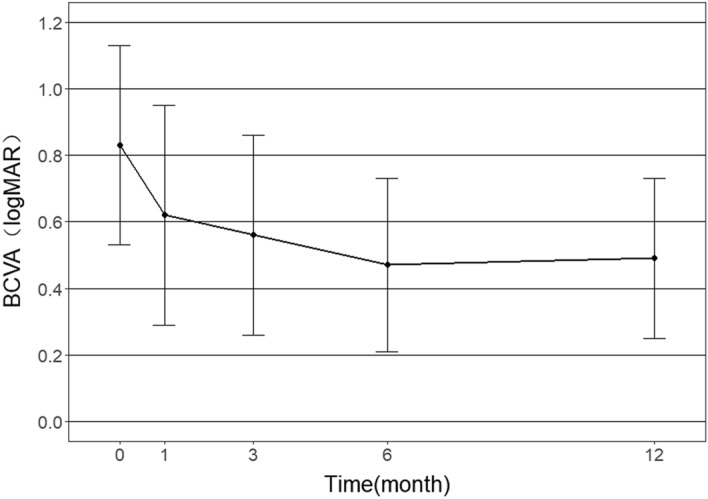
Visual acuity of mCNV eyes in patients over 50 years treated by Conbercept during one-year follow-up (N = 21).

**Figure 2:**
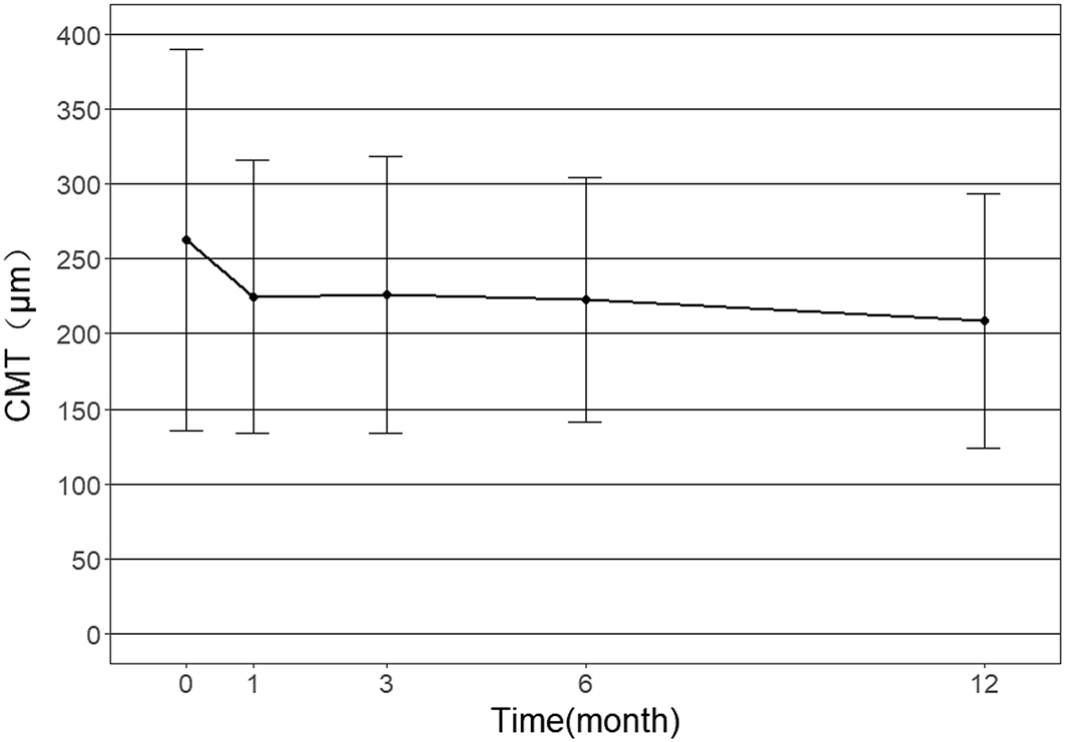
CMT of mCNV eyes in patients over 50 years treated by conbercept during one year follow-up (N = 21).

The mean BCVA improved significantly from 0.83 ± 0.30 logMAR at baseline to 0.49 ± 0.24 logMAR at the 12th month (*P* < 0.01) (Fig. 1). Repeated measures ANOVA showed significant improvements in the VA at the 1st, 3rd, 6th and 12th months compared to the baseline (*F* = 17.383, *P* < 0.001). CMT decreased from 262.86 ± 127.01 μm at baseline to 208.58 ± 85.08 μm at the 12th month (Fig. 2), but the difference was not statistically significant (*F* = 1.060, *P* = 0.352).

### Prognostic analysis of mCNV eyes in the older patients treated by Conbercept

Baseline demographic and clinical characteristics were analysed by univariate regression analysis. Baseline BCVA and ELM integrity were related to the final BCVA at the 12-month follow-up (*P* = 0.001 and 0.002, respectively), while similar results were displayed on multivariate stepwise linear regression analysis (*P* = 0.013 and 0.036, respectively). The other factors, like age, gender, axial length, et al., were filtered out in the stepwise selection procedure. (Table 3). During the one-year follow-up, the average number of injections administered was 1.71 ± 0.90 with a range of 1 to 4 (11 patients with 1 injection, 6 patients with 2 injections, 3 patients with 3 injections, and 1 patient with 4 injections).

**Table 3:** Linear regression analysis for final BCVA of mCNV patients aged over 50 years treated by Conbercept at 1-year follow-up.

Variables	Univariate		Multivariate	
	Correlation coefficient (SE value)	*P*-value (*t*-value)	Regression coefficient B (SE value)	*P* value (*t* value)
Age	− 0.095 (0.255)	0.718 (− 0.368)	–	–
Gender	0.029 (0.256)	0.911 (0.114)	–	–
Axial length	0.275 (0.246)	0.285 (1.109)	–	–
Baseline BCVA	0.738 (0.172)	0.001 (4.241)	0.517 (0.147)	0.013 (2.862)
Baseline CMT	0.298 (0.244)	0.246 (1.207)	–	–
CNV area	0.424 (0.232)	0.09 (1.814)	–	–
CNV location	− 0.274 (0.246)	0.287 (− 1.103)	–	–
IRF	0.411 (0.233)	0.101 (1.747)	–	–
SRF	− 0.362 (0.239)	0.154 (− 1.503)	–	–
SFCT	− 0.379 (0.237)	0.133 (− 1.587)	–	–
Vitreomacular abnormities	0.296 (0.244)	0.249 (1.199)	–	–
EZ integrity	0.049 (0.256)	0.853 (0.189)	–	–
ELM integrity	0.692 (0.185)	0.002 (3.711)	0.418 (0.076)	0.036 (2.314)

Adopted stepwise selection.

*BCVA* best-corrected visual acuity, *CMT* central macular thickness, *CNV* choroidal neovascularization, *ELM* external limiting membrane, *IRF* intraretinal fluid, *EZ* IS ellipsoid zone, *logMAR* logarithm of the minimum angle of resolution, *SFCT* subfoveal choroidal thickness, *SRF* subretinal fluid.

### Analysis of mCNV recurrence in older patients

**Figure 3:**
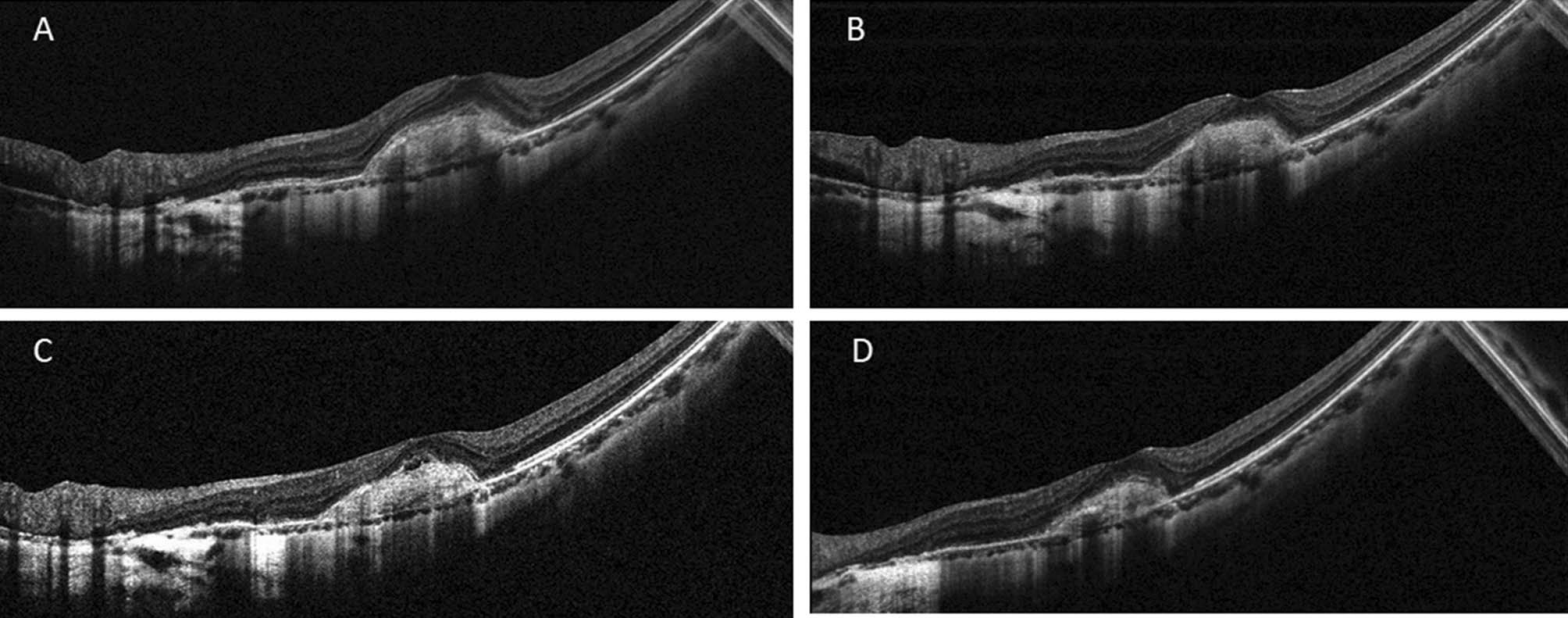
A 52-year-old male patient with mCNV. BCVA was 20/80 and CMT was 461um. (**A**) Subfoveal CNV with SRF and fuzzy border was shown on spectral-domain optical coherence tomography at baseline. (**B**) One month after 2 injections of Conbercept, subretinal fluid was absorbed and BCVA increased to 20/50. (**C**) Six month later, the patient suffered recurrence of CNV with increasing SRF and BCVA decreased to 20/80, thus one additional injection of Conbercept was given. (**D**) At one-year follow-up, CNV reduced with dry macula and vision improvement (BCVA was 20/50).

During the follow-up, 7 (33.3%) of the patients showed recurrence and the mean recurrence time was 9.40 ± 4.83 months after the first injection. The baseline central macular thickness in the recurrence group (367.57 ± 125.97 μm, Fig. 3) was greater than that in the recurrence-free group (210.50 ± 92.93 μm, *P* = 0.016). Following the reinjection of the Conbercept treatment when relapse occured, the final BCVA of patients in recurrence group was comparable to that in recurrence-free group (LogMAR: 0.49 ± 0.26 vs 0.49 ± 0.25, *P* = 0.962).

To identify the prognostic factors for mCNV recurrence in patients over 50 years, the COX proportional hazards model stepwise analysis was used and revealed that the only prognostic factor was baseline CMT (*P* = 0.019). The risk of recurrence was significantly higher in patients with CMT ≥ 262 μm compared to patients with CMT ˂ 262 μm (the average baseline CMT) (*HR* = 14). In addition, every 100-μm increment in CMT increased the risk of recurrence by 1.84 (*P* = 0.028). Other baseline factors showed no significant correlation with mCNV recurrence.

The average number of injections in the recurrence group was 2.71 ± 0.76, which was higher than that in the recurrence-free group (1.21 ± 0.43) (*t* = 5.872, *P* < 0.001). The patients were divided into two groups, taking an average CMT of 262 μm as the limit; the mean number of injections in the eyes with a greater CMT was 2.22 ± 0.97, which was greater than the 1.33 ± 0.65 times in the smaller CMT group (*t* = 2.513, *P* < 0.05).

### Adverse effects

Subconjunctiva hemorrhage were detected in four eyes after the intravitreal injection, which were absorbed in two weeks. In addition, mild cells in the anterior chamber were present in one eye, and a slightly elevated intraocular pressure in another one. All adverse effects were back to normal within two weeks after the injection. During the follow-up, no severe adverse effect was reported.

## Discussion

In this study, we first evaluated the effect of age on the severity of mCNV, and found that mCNV patients over 50 years of age had a poorer vision, thinner SFCT, and higher risk of concomitant VIA than younger patients.

Yoshida^9^ conducted a retrospective study of 63 patients with 73 eyes and the patients were divided into a younger group (age ≤ 40 years) with a mean age of 29.8 ± 5.7 years and an older group (age > 40 years) with a mean age of 54.6 ± 9.2 years. The study showed that the baseline BCVA of the younger group was better than that of the older group (0.44 ± 0.40 vs 0.89 ± 0.51), which was consistent with our results. Tabandeh^10^ included 22 mCNV patients over 50 years old with a mean age of 63.1 ± 9.8 years, and the baseline BCVA was only 1.1 ± 0.7. In addition, both studies^9,10^ indicated that during the 5-year natural course, the visual acuity of older mCNV patients was lower than that of the younger group. Therefore, age may have a significant effect on vision in the mCNV population.

Our results showed that choroidal thickness decreases with increasing age, which have also been reported in other studies^11^. In addition to the possible risk factors of the incidence of mCNV^12^, thinner choroid in older PM patients may cause further vision loss by weakening the RPE function and accelerating the progress of chorioretinal atrophy around the CNV^12^. A Chinese epidemiological study of 2044 patients^13^ showed that the incidence of vitreoretinal interface disorders was significantly correlated with age (*OR* = 1.06) and myopia (*OR* = 2.21), and another epidemiological study of 6830 patients^14^ showed similar results, which were consistent with our outcome. This means more vitreoretinal interface abnormalities have to be concerned when treating older mCNV patients.

In the Conbercept study, we found that Conbercept could significantly improve the BCVA of mCNV patients aged over 50 years after one year. Unlike wet AMD, the macular retina of eyes with mCNV was not very edematous^17^. The average CMT of mCNV patients over 50 years in our study was only 262.86 um. Therefore, it’s reasonable that no significant change in macular thickness was found even with obvious vision improvements in our study.

In our study, better baseline vision, less ELM damage, CNV location, and smaller CNV area led to better BCVA after anti-VEGF treatment. Indeed, some studies indicated the ELM visibility to be a reliable parameter for evaluating the activity of myopic CNV^15^. However, it is still controversial. In the study reported by Paolo Milani et al^21^, it was unlikely that lesion activity was correlated solely to ELM disruption and vision gain might occur despite ellipsoid zone or ELM restoration. In addition to obscuring optical reflectivity by active CNV at the level of the ELM on OCT imaging, absence of ELM visibility could be ascribed to ELM disruption which indicated an irreversible damage of the photoreceptors^18^. Some previous studies of wAMD^15^ have identified the possible role of ELM integrity in the assessment of vision prognosis. Accordingly, we presume that visibility of ELM integrity in the fovea may predict good prognosis of mCNV patients, which still needs larger sample size and longer follow-up to verify. Additionally, in the linear mixed-effects model analysis, we further found that baseline BCVA, CNV area, and CNV location associated to prognostic vision, which were reported in other studies^16–18^.

So far, increasing attentions have been gained on mCNV recurrence and its hazards to vision^25–32^. Possible risk factors related to mCNV recurrence after anti-VEGF treatment include new onset and progressive lacquer crack, age, gender, duration of myopic CNV, baseline subfoveal choroidal thickness, and CNV size. However, the conclusion is still controversial. Baseline choroidal thickness was associated with recurrence in Ahn et al.’s studies^15^, while Yang et al.^15^ reported no association between baseline SFCT and mCNV recurrence. However, young PM patients were included in these studies, while SFCT varying with different ages has been reported and accepted^15^. In our study, we would rather investigate older patients who have a higher possibility of recurrence. We found that about one third of patients had a recurrence after the Conbercept treatment, and baseline SFCT was not found to have a relationship with recurrence. Interestingly, we found that thicker macula correlated with recurrence in older mCNV patients who needed more injections (N = 2.71). In addition, when taking 262 μm as the CMT limit, the risk of recurrence was much higher (14 times) in patients with a thicker macula than in those with a thinner macula. Li et al.^16–18^ reported that thick retina was one of risk factors for mCNV patients who needed ranibizumab retreatment, in addition to age and gender. However, in their study, young patients (age < 50 years) were included and a different anti-VEGF drug was injected. We focused on older mCNV patients (age ≥ 50 years) who were treated with Conbercept and tried to assess the baseline predictors of treatment outcome and mCNV recurrence, for which very little has been published in the literature. Unfortunately, we did not evaluate the lacquer crack and its progression in our study. We will further explore the morphologic feature changes during the follow-up period in the future.

Indeed, our study has some limitations. The small number of patients was followed-up for a short period in this retrospective study, and our results have to be verified by more studies with larger sample size and longer follow-up period. Additionally, younger patients with mCNV were not enrolled as controls to evaluate the treatment efficacy of Conbercept because we mainly concerned of the older patients and some of the younger patients cannot meet the inclusive criteria in this study, thus a prospective study may be designed to evaluate the difference efficacy of Conbercept between the younger group and the older group in the future.

In conclusion, our study showed that older mCNV patients (over 50 years old) had worse vison, thinner choroid, and increased vitreoretinal interface abnormalities than younger patients. In addition, baseline BCVA, ELM, CNV lesion size, and CNV lesion location were correlated with BCVA after one year of Conbercept therapy. Baseline CMT may be a predictor of relapse in older mCNV patients during the one-year follow-up. Therefore, more attention has to be given to patients with a thicker macula at baseline, since it was one of the risk factors for recurrence and the patients may need more retreatment. This still needs further investigation.

## Data Availability

The datasets generated during and/or analyzed during the current study are available from the corresponding author on reasonable request.
